# CD98hc, a novel of galectin-8 receptor, binds to galectin-8 in an N-glycosylation-dependent manner: CD98hc is a new binding partner for Gal-8

**DOI:** 10.3724/abbs.2024182

**Published:** 2025-01-07

**Authors:** Yunlong Si, Jiahui Zhu, Hend Sayed, Kevin H. Mayo, Yifa Zhou, Guihua Tai, Jiyong Su

**Affiliations:** 1 Jilin Province Key Laboratory for Chemistry and Biology of Natural Drugs in Changbai Mountain School of Life Sciences Northeast Normal University Changchun 130024 China; 2 Jiangsu Key Laboratory of Brain Disease Bioinformation Research Center for Biochemistry and Molecular Biology Xuzhou Medical University Xuzhou 221004 China; 3 Department of Biochemistry Molecular Biology & Biophysics 6-155 Jackson Hall University of Minnesota 321 Church Street Minneapolis MN 55455 USA.

**Keywords:** galectin-8, CD98hc, *N*-glycosylation, microscale thermophoresis

## Abstract

Glycan-mediated recognition plays a critical role in facilitating cell-cell and cell-matrix interactions. Galectin-8 (Gal-8), classified as a ‘tandem-repeat’ type of galectin, binds to cell surface glycans to modulate various cellular functions, including cell adhesion, migration, apoptosis, pathogen recognition, autophagy, and immunomodulation. Despite the known function of Gal-8 in binding to various glycosylated proteins, only a few interactions have been reported to date. In this study, mass spectrometry is used to identify CD98hc as a novel binding partner for Gal-8. Both the N-terminal and C-terminal carbohydrate recognition domains (CRDs) of Gal-8 (Gal-8N and Gal-8C) bind to CD98hc, an interaction that is specifically inhibited by lactose but not sucrose, as confirmed by pull-down assays. The binding affinity between CD98hc and Gal-8 measured by microscale thermophoresis (MST) is 1.51 ± 0.17 μM. In addition, Gal-8N and Gal-8C have the binding affinities of 0.22 ± 0.03 μM and 10.68 ± 1.69 μM, respectively. Gal-8N and Gal-8C are both involved in the recognition and binding process of CD98hc. Furthermore, both full-length Gal-8 and its individual CRDs bind specifically to *N*-glycosylated glycans on CD98hc, as demonstrated by the use of tunicamycin to inhibit *N*-glycosylation in cells. In addition, Gal-8 and its individual CRDs can pull down glycosylated CD98hc-ED but not free CD98hc-ED *in vitro*, indicating that the binding of Gal-8 to glycosylated CD98hc-ED is *N*-glycosylation-dependent. Overall, our findings establish CD98hc as a novel binding partner for Gal-8 and provide insights for further exploration of the diverse biological functions of Gal-8.

## Introduction

Protein glycosylation, which is crucial to cell and tissue physiology, is highly complex and regulates numerous pathological disorders, and interactions between various cells and their environments are partially dependent on glycosylation [1]. Galectin-8 (Gal-8) belongs to a family of lectins that generally bind to β-galactoside-based glycoconjugates [ 2– 4] . Galectins are generally divided into three groups: (i) prototype galectins (Gal-1, -2, -5, -7, -10, -11, -13, -14, -15 and -16) with a single carbohydrate recognition domain (CRD), (ii) tandem-repeat galectins (Gal-4, -6, -8, -9, and -12) with two different CRDs joined by a linker peptide, and (iii) chimera-type Gal-3 with a single CRD linked to a long, aperiodic and proline-rich N-terminal tail [ 4– 6] . As a tandem-repeat galectin, Gal-8 is composed of N- and C-CRDs connected by a peptide linker of variable length [ 7– 9] . The N- and C-CRDs of Gal-8 are approximately 35% homologous, whereas their glycan specificities differ significantly. This bifunctionality makes it possible for Gal-8 to cross-link a wide variety of glycoconjugates, contributing to the functional diversity of Gal-8 [ 10, 11] .

Compared with other tandem-repeat galectins, Gal-8 is more widely distributed in mammalian tissues, such as the liver, heart, muscles, kidney, and brain [12]. Gal-8 lacks a signal peptide and is thus secreted extracellularly via a nonclassical pathway. This process involves Gal-8 accumulating on the cytoplasmic side of the plasma membrane and being released in vesicles and exosomes or translocating directly through the plasma membrane [13]. In the extracellular space, Gal-8 binds to cell surface glycoproteins that trigger a downstream signaling cascade with various receptors, such as FAK, ERK, and JNK [12]. This, in turn, modulates various cellular functions, from cell adhesion, migration, and apoptosis to pathogen recognition, autophagy, and immunomodulation [ 12, 14, 15] . These findings underscore the biological importance of Gal-8.

As a major class of cell surface adhesion receptors, integrins (and other adhesion receptors) bind to Gal-8 to promote cell adhesion [ 16– 19] . Several of these receptors are known. For example, CD44vRA is a variant of CD44 that has high affinity for Gal-8. In rheumatoid arthritis (RA), the formation of this complex influences the extent of the autoimmune inflammatory response [20]. CD166, also known as activated leukocyte cell adhesion molecule (ALCAM), is overexpressed in various tumors, and its expression level and cell surface abundance are correlated with poor prognosis [21]. CD166 is a Gal-8-binding partner involved in the regulation of cell adhesion and migration of tumor cells, as well as endothelial cell-based angiogenesis, processes that are critical to tumor progression [ 22, 23] . Podoplanin (PDPN) is a heavily *O*-glycosylated, small mucin-type transmembrane glycoprotein with a wide variety of functions, including the regulation of cell motility and adhesion [24]. Cueni and Detmar [25] reported that Gal-8 binds to PDPN to promote the adhesion and haptotaxis of lymphatic endothelial cells (LECs). In addition, Gal-8 interacts with PDPN to promote lymphangiogenesis and cancer cell migration (metastasis) through the lymphatic system [ 24, 26] . CD45 is a transmembrane PTPase that primarily targets the Src kinases Lck and Fyn. Because Lck, in particular, is a primary initiator of signal transduction upon TCR engagement, its absence can lead to severe disruption of T-cell differentiation and impaired activation of mature T cells [27]. Campetella *et al*. [28] reported that Gal-8 acts as a T-cell activator by agonistically binding to CD45. Coagulation factor V (FV) serves as a cofactor in the prothrombinase complex, activating factor X (FXa), and thus plays a critical role in the operation of the coagulation cascade [28]. Gal-8 interacts with FV in a carbohydrate-dependent manner to mediate FV endocytosis and thus regulates platelet function [29]. In the cytoplasm, Gal-8 acts as a “danger receptor” that recruits NDP52, a protein reported to restrict *S*. *typhimurium* proliferation and direct autophagy with invading bacteria [ 15, 30, 31] .

Although the binding of Gal-8 to these receptors mediates significant biological effects, much remains unknown about how Gal-8 works mechanistically at the molecular level. One way to expand our understanding of the different functions of Gal-8 is to identify specific Gal-8-binding glycoproteins. In the present study, using biochemical and cell-based techniques, we demonstrated that Gal-8 binds to *N*-glycans on CD98hc. CD98hc (also known as 4F2hc, FRP-1 or SLC3A2) is the heavy chain (hc) of an *N*-glycosylated membrane protein in the family of heterodimeric amino acid transporters that regulate amino acid transport and intracellular integrin signaling. In this context, they play important roles in placental development, tumorigenesis, and immune homeostasis [ 32– 35] . CD98hc is also a target for the delivery of biotherapeutics in the brain [36]. Here, we identified CD98hc as a novel Gal-8 receptor, providing further insight into Gal-8-mediated functions.

## Materials and Methods

### Cloning, overexpression and purification of recombinant proteins

Recombinant Gal-8, Gal-8N and Gal-8C were expressed in *Escherichia coli* BL21 (DE3) pLysS strains as previously described by Si *et al*. [9]. Gal-8-eGFP was generated using the following primers: 5′-GGATCCCATATGATGCTGAGCCTGAAC-3′ and 5′-GGATCCGGATCCCCAACTACGAACTTC-3′. Gal-8N-eGFP was generated using the following primers: 5′-GGATCCCATATGATGCTGAGCCTGAAC-3′ and 5′-GGATCCGGATCCGCTGAAACCGATGCT-3′. Gal-8C-eGFP was generated using the following primers: 5′-GGATCCCATATGTTTGCAGCACGTCTG-3′ and 5′-GGATCCGGATCCCCAACTACGAACTTC-3′. After digestion with the *Nde*I and *Bam*HI restriction enzymes, the PCR products were cloned and inserted into the pET28a-eGFP-C vector, which was confirmed by DNA sequencing. Recombinant proteins were expressed in *E*. *coli* BL21 (DE3) bacteria, and purified on a Ni-NTA agarose column (Qiagen, Hilden, Germany) according to previous studies [ 8, 9, 37] .

### GFP pull-down and mass spectrometry

GFP pull-down was performed as described previously [ 38, 39] with some modifications. Briefly, 100 μg of Gal-8-eGFP and 300 μg of HeLa cell lysate were incubated with 50 μL of GFP beads (CH10001; Jilin Province Chenghe Trade, Changchun, China) overnight at 4°C with constant mixing. The beads were then washed twice with ice-cold Tris buffer, collected and boiled in SDS/PAGE buffer, and then analyzed by SDS-PAGE. Protein identification was performed by LC-MS/MS peptide sequencing, with analysis performed using the ProtTech software (Norristown, USA).

### His-tag pull-down and western blot analysis

The methods used for His-tag pull-down and western blot analysis were described in our previous papers [ 38– 40] . Gal-8N and Gal-8C were also assessed by pull-down experiments, and the effects of lactose and sucrose were also assessed.

### Cloning and expression of the extracellular domain of CD98hc (CD98hc-ED)

Total RNA was extracted using Trizol (Thermo Fisher Scientific, Waltham, USA) from HeLa cells and then reverse transcribed using a reverse transcription kit (Takara, Dalian, China) according to the manufacturer’s instructions. Amplified cDNA was used as a template for subsequent amplification of CD98hc. The DNA for CD98hc-ED with a His-tag was generated using the following primers: 5′-GGATCCAAGCTTATGCGAGCGCCGCGTTGTCGC-3′ and 5′-GGATCCCTCGAGTTAGTGGTGGTGGTGGTGGTGGGCCGCGTAGGGGAAGCG-3′. After digestion with *Hin*dIII and *Xho*I restriction enzymes, the PCR products were cloned and inserted into a pCDNA3.1(+) vector, which was confirmed by DNA sequencing.

HEK293F cells were cultured in SMM 293T-I medium (Sino Biological, Inc., Shanghai, China) using a Multitron-Pro shaker. When the cell density reached 2 × 10 ^6^ cells/mL, the pCDNA3.1(+)-CD98hc-ED plasmid was transfected into the cells. Transfected HEK293F cells were harvested and sonicated on ice. After sonication, the suspension was centrifuged, and the supernatant was incubated with Ni-NTA agarose. Prepurified CD98hc-ED was concentrated using a 30-kDa cut-off Centricon (Millipore, Billerica, USA) and further purified by size exclusion chromatography (Superdex 200 10/300 GL; GE Healthcare, Wisconsin, USA). The peak fractions were pooled and concentrated to ~1 mg/mL. The expression of CD98hc-ED was also analyzed using a dot-blot assay. Briefly, 1 μL of samples (fractions from size exclusion chromatography column, SEC) were applied over nitrocellulose membranes (GE Healthcare Life Sciences, Pittsburgh, USA) and air dried at room temperature. Membrane was blocked with 5% nonfat dry milk in PBST (PBS containing 0.05% Tween-20) for 1 h and incubated with CD98hc antibody overnight at 4°C, followed by incubation with HRP-conjugated goat anti-rabbit IgG after washing thrice with PBST. All the protein blots were developed by ECL Plus Western Blotting Detection kit (Solarbio, Beijing, China).

### MicroScale thermophoresis (MST)

The MST assay was performed as previously reported [ 38, 39] . Briefly, His-tagged CD98hc-ED (50 nM) used as a target was labelled with a fluorescence dye kit, RED-tris-NTA 2 ^nd^ generation (NanoTemper Technologies, Munich, Germany), and nonfluorescent Gal-8 was titrated in a 1:1 dilution series between 50 μM and 12.21 nM. The samples were measured using Monolith NT.115 (NanoTemper Technologies) at room temperature setting with LED/excitation power of 60% and MST power of medium. The data were analyzed using MO Affinity Analysis Software *K*_D_ fit (version 2.2.5; NanoTemper Technologies). Gal-8N and Gal-8C were also assessed by MST assay. Three independent experiments were performed.

### Protein deglycosylation

HeLa cells were treated with tunicamycin (Beyotime, Shanghai, China), an *N*-linked glycosylation inhibitor (1 μg/mL, 5 μg/mL or 10 μg/mL), for 48 h in 6-cm plates in DMEM. Cell lysates were used to assess CD98hc expression, and interactions between deglycosylated CD98hc and Gal-8 were determined by western blot analysis. The levels of Gal-8N and Gal-8C were also determined via a protein deglycosylation assay.

### Gal-8 pulldown of glycosylated and free CD98hc-ED

Pull-down assays were performed using glycosylated and deglycosylated forms of CD98hc-ED. Glycosylated CD98hc-ED was obtained as described in the section of “Cloning and expression of the extracellular domain of CD98hc (CD98hc-ED)”. Free CD98hc-ED is obtained by the enzymatic cleavage of *N*-linked oligosaccharides from glycosylated CD98hc-ED using N-glycosidase F (PNGase F), a commonly used enzyme for the deglycosylation of glycoproteins. For each microgram of glycosylated CD98hc-ED, 5 units of PNGase F (G3468; Servicebio Biotechnology, Wuhan, China,) were used in a 16-h reaction on ice to ensure the complete removal of *N*-linked oligosaccharides, as confirmed by western blot analysis. Subsequently, 100 μg of Gal-8-eGFP was incubated overnight at 4°C with constant mixing with either 100 μg of glycosylated or deglycosylated CD98hc-ED in 50 μL of GFP beads. After incubation, the beads were washed twice with ice-cold Tris buffer, collected, boiled in SDS/PAGE buffer, and subjected to western blot analysis. The interaction between Gal-8N-eGFP and Gal-8C-eGFP was also investigated under similar conditions.

## Results

### Protein isolation and identification

**Figure 1:**
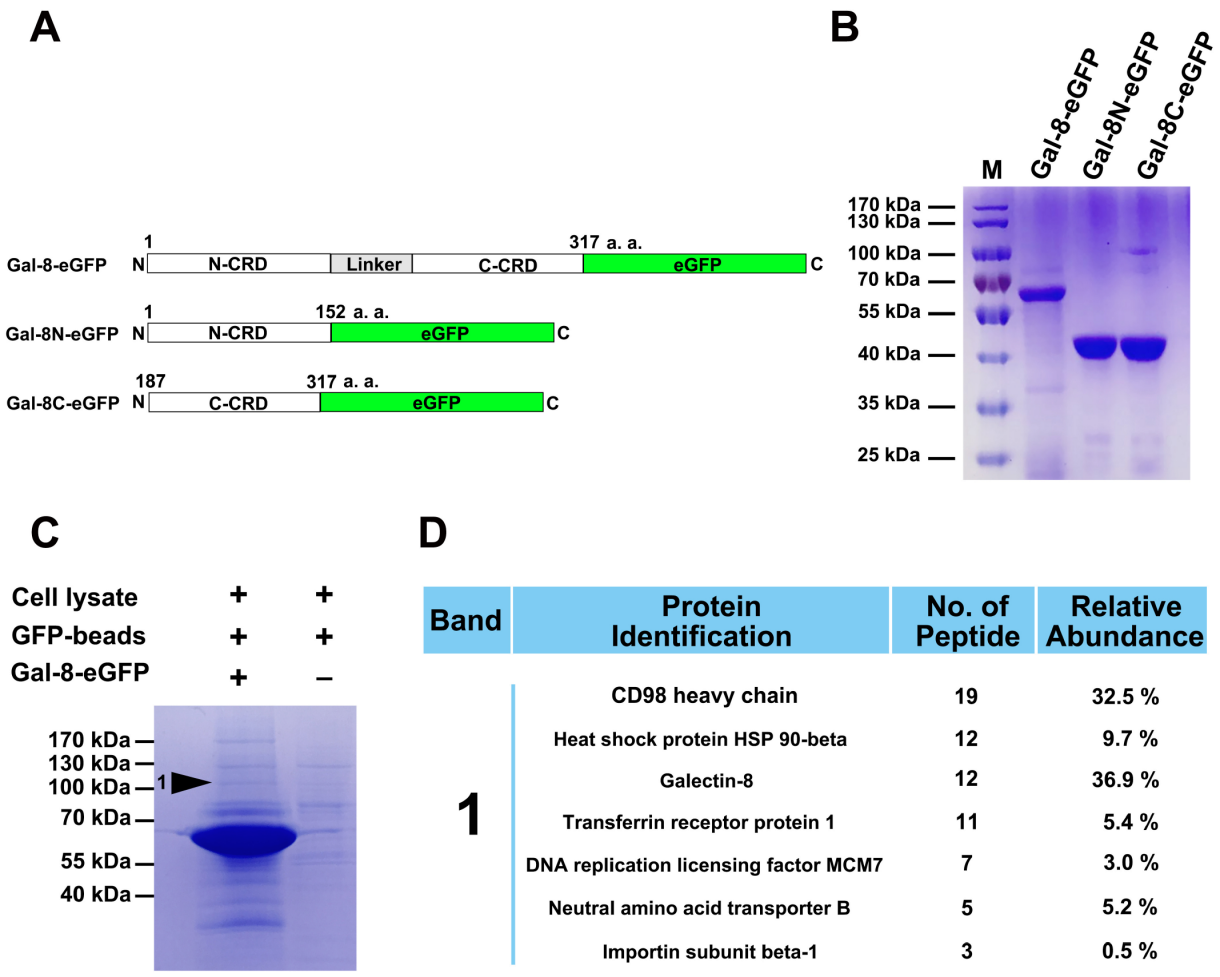
Figure 1 Characterization of Gal-8-interacting proteins (A) A schematic diagram of Gal-8-eGFP, Gal-8N-eGFP and Gal-8C-eGFP. (B) SDS-PAGE analysis of the purities of Gal-8-eGFP, Gal-8N-eGFP and Gal-8C-eGFP. (C) SDS-PAGE of the proteins pulled down by Gal-8. The specific protein band marked 1 was subjected to mass spectrometry. (D) List of the proteins identified by LC-MS/MS. CD98hc is a potential Gal-8-binding protein.

To search for novel Gal-8-interacting proteins, we first generated Gal-8-eGFP ( Figure 1A,B) and used GFP beads (which have high affinity for Gal-8-eGFP) to pull down associated proteins in HeLa lysates. The negative control contained GFP beads and HeLa lysate without Gal-8-eGFP. The protein profile of the Gal-8-eGFP pull-down HeLa lysate was determined by Coomassie blue staining ( Figure 1C). As expected, one distinct band at ~110 kDa was analyzed by LC-MS/MS peptide sequencing. This approach identified the proteins with >99.9% certainty ( Figure 1D).

The molecular weight (MW) of CD98hc is 120–130 kDa, which is similar to the estimated MW. In addition, 19 short peptides of CD98hc were observed ( Supplementary Data S1). On the basis of the characteristics of these proteins, subsequent studies focused on CD98hc.

### CD98hc is a binding partner of Gal-8

**Figure 2:**
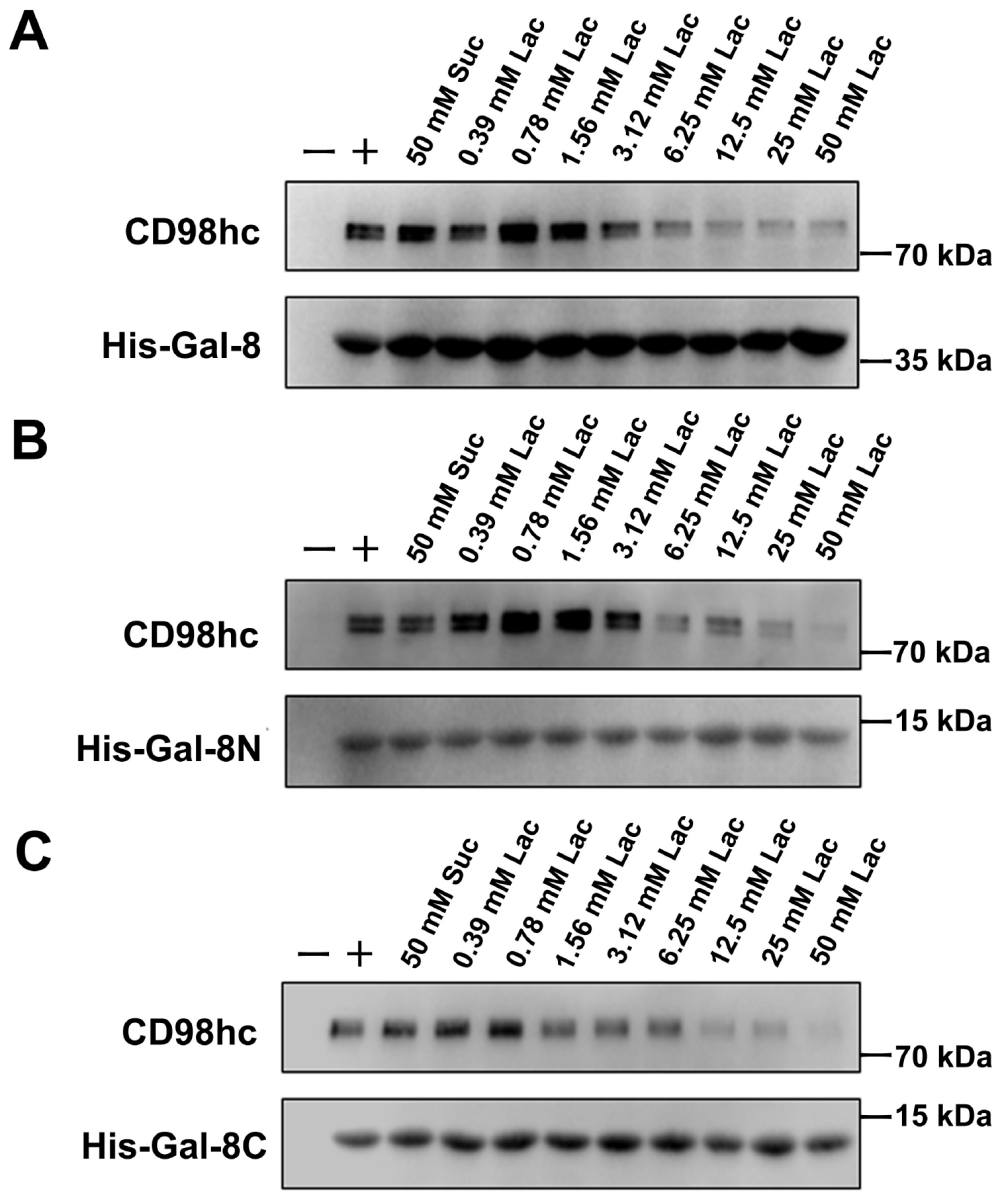
Figure 2 CD98hc interacts with Gal-8, Gal-8 N, and Gal-8C (A) Gal-8 effectively pulled down CD98hc, and this interaction was inhibited by lactose but not by sucrose. As the concentration of lactose increased, the inhibitory effect became more pronounced. (B) Gal-8N also demonstrated the ability to pull down CD98hc, and this interaction was similarly inhibited by lactose but not by sucrose. As the concentration of lactose increased, the inhibitory effect became more pronounced. (C) Gal-8C interacted with CD98hc, which was inhibited by lactose but not by sucrose. As the concentration of lactose increased, the inhibitory effect became more pronounced. The CD98hc antibody was exposed to CD98hc, and the His-tag antibodies were exposed to His-Gal-8, His-Gal-8N and His-Gal-8C.

To confirm that Gal-8 binds to CD98hc, we first used pull-down assays. Gal-8 can pull down CD98hc, indicating that Gal-8 binds to CD98hc. To demonstrate that Gal-8/CD98hc binding is β-galactoside-dependent, we added lactose (a competitive inhibitor of galectin binding) to the mixture and observed that this disaccharide indeed inhibited the interaction, whereas the negative control sucrose (50 mM) did not ( Figure 2A). These findings show that Gal-8 interacts with CD98hc in a carbohydrate-dependent manner. Gal-8 is a tandem-repeat galectin that has two different CRDs (Gal-8N and Gal-8C) joined by a linker peptide. We found that both Gal-8N and Gal-8C can also pull down CD98hc. As with Gal-8, the binding of both Gal-8N and Gal-8C to CD98hc is inhibited by lactose but not by sucrose even at 50 mM ( Figure 2B,C). These findings indicate that both Gal-8N and Gal-8C interact with CD98hc in a β-galactoside-dependent manner.

### Gal-8 binds to CD98hc with high affinity

**Figure 3:**
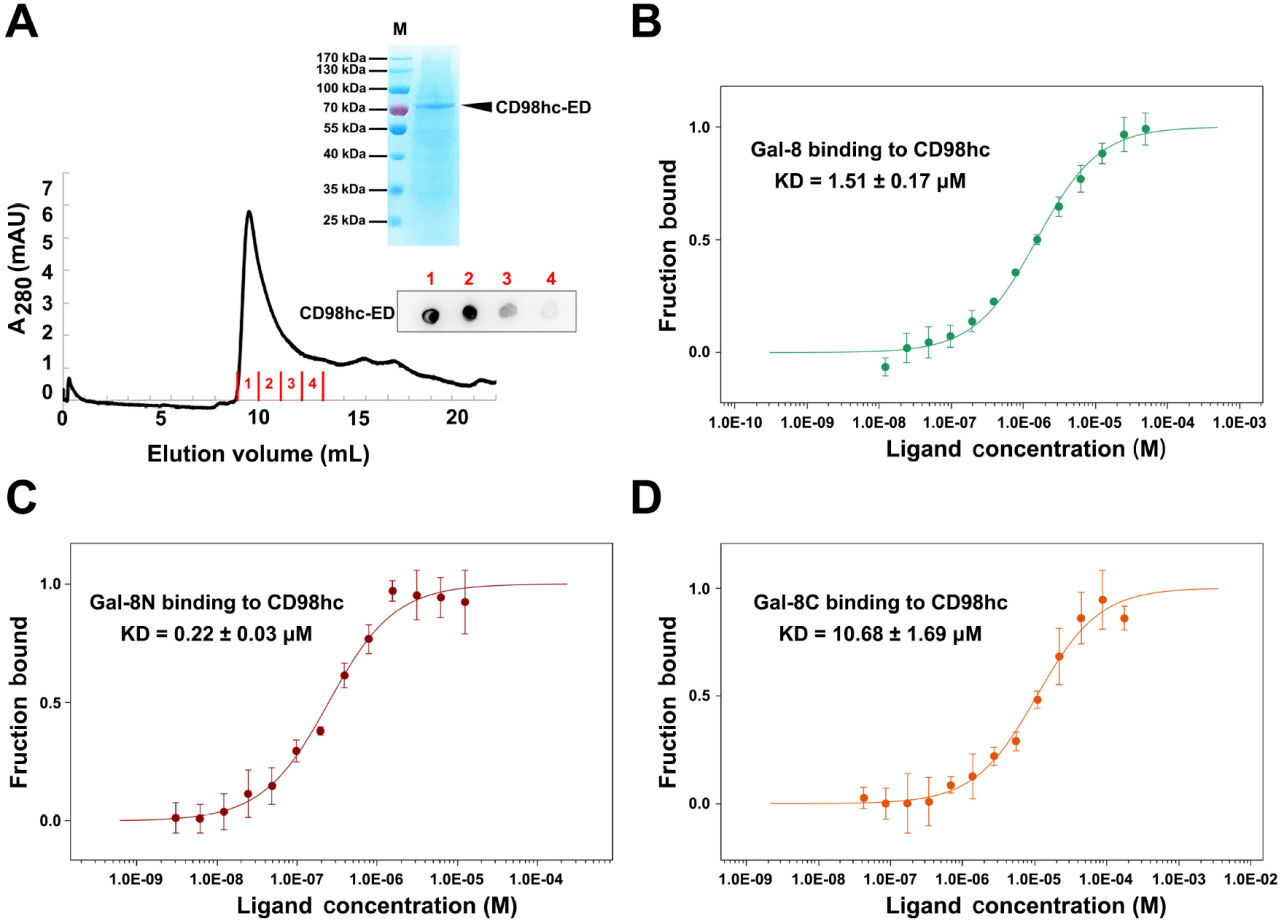
Figure 3 Binding affinities of CD98hc-ED for Gal-8, Gal-8N, and Gal-8C determined by MST assay (A) Gel filtration, SDS-PAGE, and dot-blot assays were employed for the purification of CD98hc-ED. The glycosylated form of CD98hc-ED was detected using a specific CD98hc antibody via a dot-blot assay. (B) MST was used to assess the binding affinity of Gal-8 for CD98hc-ED. (C) MST analysis was performed to measure the binding affinity of Gal-8N for CD98hc-ED. (D) MST experiments were conducted to determine the binding affinity of Gal-8C for CD98hc-ED. Here, the fraction bound is plotted against the ligand concentration. All ΔRatio (Spectral Shift) or ΔFnorm (MST) values of a curve are divided by the curve amplitude, resulting in the fraction bound (from 0 to 1) for each data point. The error bars represent the standard deviations (n = 3).

Both Gal-8 and CD98hc bind to β1 integrin; thus, an indirect interaction between Gal-8 and CD98hc is always possible. To confirm the direct interaction between Gal-8 and CD98hc, we expressed and purified the extracellular domain (ED) of CD98hc (CD98hc-ED) and assessed its binding affinity for Gal-8 by MST assay. CD98hc-ED was expressed in HEK293F cells and purified by Ni-NTA affinity and size exclusion chromatography. The purity of CD98hc-ED was determined by SDS-PAGE (> 90%) ( Figure 3A). The binding affinities of Gal-8, Gal-8N and Gal-8C to CD98hc-ED were examined. Here, we found that Gal-8 has a high affinity for CD98hc-ED, with a value of 1.51 ± 0.17 μM. Furthermore, compared with Gal-8, Gal-8N has a greater affinity ( *K*_D_ of 0.22 ± 0.03 μM), whereas Gal-8C also has a strong affinity ( *K*_D_ of 10.68 ± 1.69 μM) for CD98hc-ED ( Figure 3B‒D). Therefore, the affinity of Gal-8 binding to CD98hc is actually the cumulative affinity of Gal-8N and Gal-8C, indicating that Gal-8N and Gal-8C collectively participate in the recognition and binding process of CD98hc. These results demonstrate that Gal-8, Gal-8N and Gal-8C all directly interact with CD98hc and that Gal-8N and Gal-8C are both involved in the recognition and binding process of CD98hc.

### Gal-8 binds to N-glycosylation sites on CD98hc

**Figure 4:**
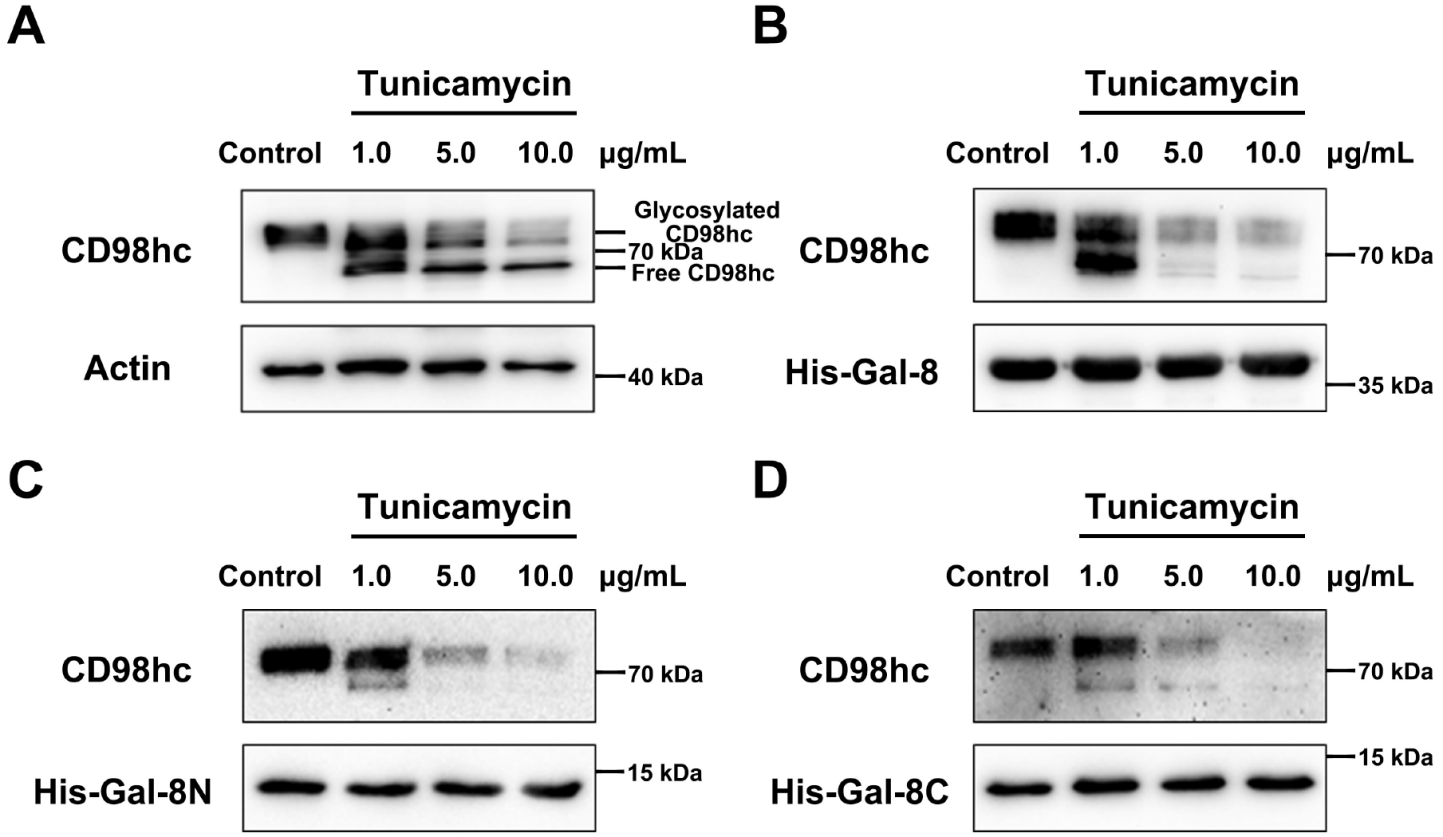
Figure 4 CD98hc does not interact with Gal-8, Gal-8N, or Gal-8C after treatment with tunicamycin (A) Tunicamycin effectively inhibits the N-glycosylation of CD98hc. The molecular weight of glycosylated CD98hc is noticeably greater than that of its deglycosylated form. (B) Tunicamycin inhibits CD98hc pulled down by Gal-8. (C) Tunicamycin inhibits CD98hc pulled down by Gal-8N. (D) Tunicamycin inhibits CD98hc pulled down by Gal-8C. The CD98hc antibody was exposed to CD98hc, and the His-tag antibodies were exposed to His-Gal-8, His-Gal-8N and His-Gal-8C.

CD98hc is an *N*-glycosylated transmembrane protein receptor. Our present study has already shown that Gal-8 binds to CD98hc in a β-galactoside-dependent manner. Here, we show that these CD98hc saccharides are N-glycosylated. For this purpose, we pretreated HeLa cells with tunicamycin, which inhibits N-glycosylation on CD98hc, and used a pull-down assay with Gal-8, Gal-8N and Gal-8C. N-glycosylation of CD98hc was completely inhibited by 10 μg/mL tunicamycin. In this state, CD98hc could no longer bind to Gal-8, Gal-8N or Gal-8C ( Figure 4), thus demonstrating that Gal-8 binds to *N*-glycosylated glycans on CD98hc.

**Figure 5:**
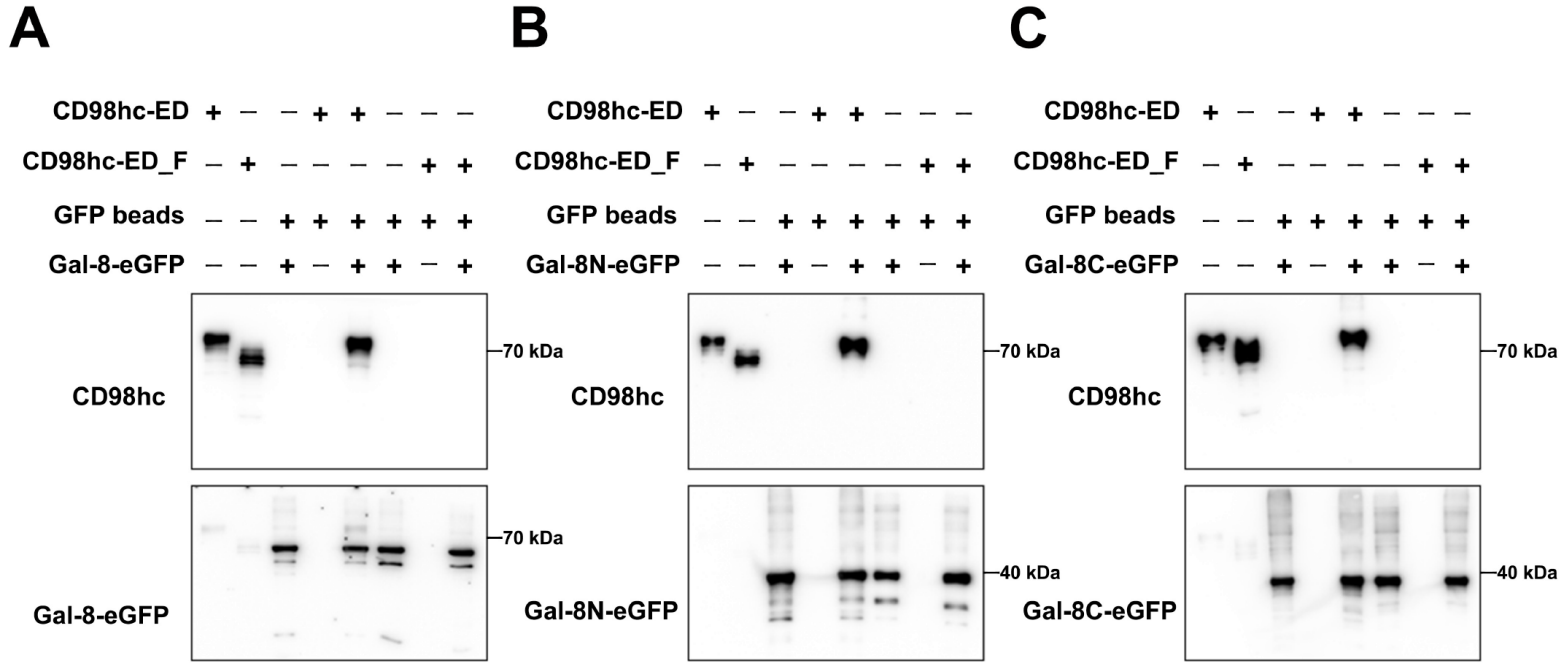
Figure 5 Pull-down of glycosylated CD98hc-ED and free CD98hc-ED by Gal-8, Gal-8N and Gal-8C (A) Lane 1 represents glycosylated CD98hc-ED, and lane 2 represents free CD98hc-ED. Gal-8 could pull down glycosylated CD98hc-ED but not free CD98hc-ED. (B). Lane 1 represents glycosylated CD98hc-ED, and lane 2 represents free CD98hc-ED. Gal-8N could pull down glycosylated CD98hc-ED but not free CD98hc-ED. (C) Lane 1 represents glycosylated CD98hc-ED, and lane 2 represents free CD98hc-ED. Gal-8C could pull down glycosylated CD98hc-ED but not free CD98hc-ED. CD98hc-ED indicates glycosylated CD98hc-ED, and CD98hc-ED_F indicates free CD98hc-ED after PNGase_F treatment. Exposure of the anti-Gal-8 antibody to Gal-8-eGFP and Gal-8N-eGFP and exposure of the eGFP antibody to Gal-8C-eGFP. “+” indicates that the band contains the substance, whereas “‒” indicates that the band does not contain the substance.

To further confirm the N-glycosylation dependency of the interaction between Gal-8 and CD98hc, an *in vitro* pull-down assay was conducted to compare the binding affinities of glycosylated and deglycosylated CD98hc-ED with those of Gal-8. Free CD98hc-ED was obtained by treating glycosylated CD98hc-ED with PNGase F, resulting in a significant decrease in molecular weight, indicating successful deglycosylation. A pull-down assay demonstrated that Gal-8 could effectively pull down glycosylated CD98hc-ED but not free CD98hc-ED, highlighting the N-glycosylation dependency of Gal-8 binding to CD98hc ( Figure 5A). Similarly, Gal-8N and Gal-8C were able to pull down glycosylated CD98hc-ED but did not interact with free CD98hc-ED ( Figure 5B,C). These findings strongly support and confirm that CD98hc is a novel glycosylated ligand of Gal-8 and that the interaction is dependent upon the N-glycosylation status of CD98hc.

## Discussion

Binding of Gal-8 to various protein receptors facilitates several important biological functions, including cell adhesion, migration, apoptosis, pathogen recognition, autophagy, and immunomodulation. [ 12, 14, 15, 41] . These functions are generally involved in various pathological disorders, such as Alzheimer’s disease, osteoarthritis, multiple sclerosis, and tumors [ 42– 45] . However, the mechanism by which Gal-8 functions is not fully understood. Therefore, we proposed an effective strategy to explore some novel functions of Gal-8 and to identify new protein ligands of Gal-8.

Here, we showed that CD98hc is a novel glycosylated ligand for Gal-8 that binds to N-glycosylated sites on CD98hc. CD98hc is a type II transmembrane N-glycosylated membrane protein that functions both as a co-receptor of β-integrins to increase intracellular downstream signaling and as a transporter of branched-chain and aromatic amino acids [ 34, 46, 47] . A growing body of evidence has shown that CD98hc is overexpressed in various cancer cells and promotes malignant cell transformation and progression [ 46, 48] . Notably, Gal-8 has also been shown to bind to and activate selected β1-integrins in different cellular systems [ 16, 49, 50] . Therefore, Gal-8 promotes downstream signaling via direct interactions with β-integrins and by binding to CD98h. Moreover, the amino acid transport function of CD98hc has been suggested to play crucial roles in the growth, proliferation and survival of cancer cells, as well as in metastasis [51]. Rapidly proliferating cells undergo extensive metabolic reprogramming to meet their increased demand for amino acids which are not only used for protein synthesis but also serve as nitrogen and carbon sources for the synthesis of nucleotides, amino sugars and glutathione [ 52– 54] . However, we discovered that Gal-8 plays a role in regulating cellular amino acid transport by binding to CD98hc, thus providing a foundation for future research into the function of Gal-8 in regulating amino acid transport.

Gal-3, the only chimera-type galectin, has been reported to bind to CD98hc and regulate tumorigenicity in HeLa cells [55]. Gal-3 binding to CD98hc can also regulate alternative macrophage activation, Bewo cell fusion, and atrial arrhythmogenesis [ 56, 57] . On the other hand, Gal-8 is a tandem-repeat galectin that has two different CRDs joined by a linker peptide. Unlike other galectins, the N-terminal CRD of Gal-8 (Gal-8N) displays a unique specificity for α2,3-sialylated glycans [ 58, 59] . Here, we discovered that Gal-8 binds to CD98hc in an *N*-glycosylation-dependent manner, with Gal-8N showing a higher affinity for CD98hc than Gal-8C does. When fitting the Gal-8 binding curve to CD98hc, we utilized the *K*d model instead of the Hill model. One reason for this is that although both Gal-8N and Gal-8C bind to CD98hc, their affinities do not differ significantly, being less than two orders of magnitude. Additionally, the binding curve of Gal-8 with CD98hc that we obtained exhibited a standard “S” shape rather than a biphasic binding curve. As reported by Lifeng Pan [60], therefore, we chose to fit using the *K*d model. The measured affinity of Gal-8 binding to CD98hc falls between that of Gal-8N and Gal-8C. Hence, the affinity of Gal-8 binding to CD98hc actually represents the cumulative affinity of Gal-8N and Gal-8C, indicating that Gal-8N and Gal-8C collectively participate in the recognition and binding process of CD98hc. The binding epitopes between Gal-8 and CD98hc may differ from those between Gal-3 and CD98hc, and this, in turn, may impact their individual biological functions. The crystal structure of CD98hc complexed with L-type amino acid transporter 1 (LAT1) (PDB:6IRT, PDB:6JMQ) revealed that CD98hc-ED has four *N*-glycosylation sites (Asn 365, Asn 381, Asn 424 and Asn 508), each of which contains *N*-acetylglucosamine residues (GlcNAc, NAG). In addition, all exposed glycans are located on the distal part of the CD98hc-LAT1 interaction interface, which is not involved in the formation of CD98hc-LAT1 heterodimers [ 61, 62] . This finding suggests that Gal-8 may bind to the *N*-glycans on CD98hc, a hypothesis that our laboratory is currently investigating.

In conclusion, we showed here that Gal-8 binds to CD98hc, whereas both Gal-8N and Gal-8C bind to CD98hc, Gal-8N dominates this interaction. In addition, we demonstrated that Gal-8 binds to *N*-glycosylated sites on CD98hc. CD98hc is a transporter of branched-chain and aromatic amino acids, suggesting that Gal-8 binding to CD98hc may be involved in cellular amino acid transport, which is essential for maintaining cellular metabolism and physiological functions. Our findings provide further insight into the cellular functions of human Gal-8.

## Supporting information

Supplementary_data_1

## Supplementary Data

Supplementary data is available at *Acta Biochimica et Biophysica Sinica* online.
